# Response rate and safety in patients with hepatocellular carcinoma treated with transarterial chemoembolization using 40-µm doxorubicin-eluting microspheres

**DOI:** 10.1007/s00432-020-03370-z

**Published:** 2020-09-02

**Authors:** Katharina Carolin Albrecht, René Aschenbach, Ioannis Diamantis, Niklas Eckardt, Ulf Teichgräber

**Affiliations:** Institut für Diagnostische und Interventionelle Radiologie, Universitätsklinikum Jena IDIR, Am Klinikum 1, 07747 Jena, Germany

**Keywords:** Transarterial chemoembolization, TACE, Hepatocellular carcinoma, Doxorubicin-eluting microspheres

## Abstract

**Purpose:**

To evaluate the response rate and safety of superselective drug-eluting beats transarterial chemoembolization (DEB-TACE) with doxorubicin-loaded 40-µm microspheres in patients with hepatocellular carcinoma (HCC).

**Methods:**

One hundred and forty-one treatments with doxorubicin-loaded 40-µm microspheres in 83 patients between 2012 and 2017 were retrospectively evaluated. Images of the treated lesions were analyzed before and after each treatment according to mRECIST (modified Response Evaluation Criteria in Solid Tumors). Therapy response (complete response [CR] + partial response [PR]) and disease control (CR + PR + stable disease [SD]) rates were determined, and the correlation between the longitudinal axis (longest diameter of the tumor) and volume was investigated using a newly developed software for systematic tumor response assessment. Additional endpoints were progression-free survival (PFS) and time to progression (TTP).

**Results:**

In the target tumors, a therapy response rate of 63.1% and a disease control rate of 95.7% were achieved. There was a good correlation between the measurement of the longitudinal axis and volume of the measured lesion (*r* value, 0.954). The median PFS was 2.23 months, and the median TTP was 5.91 months. The serious adverse event rate (SAE) was 10.64%.

**Conclusion:**

Superselective DEB-TACE with 40-µm sized Embozene Tandem™ can be considered an effective and safe treatment, given the number of procedure-related complications.

**Electronic supplementary material:**

The online version of this article (10.1007/s00432-020-03370-z) contains supplementary material, which is available to authorized users.

## Introduction

The current standard treatment for early and intermediate-stage hepatocellular carcinoma (HCC) is transarterial chemoembolization (TACE) (Lammer et al. 2010). Nearly half of all patients with HCC receive TACE at some time point during the course of their disease (Lencioni et al. 2014). TACE is a multifaceted treatment including several different technical approaches. Conventional TACE was the first established method. The technique uses lipiodol mixed with antineoplastic emulsion (Han et al. 2014). Two randomized controlled trials have illustrated the survival benefits of conventional TACE over supportive care (Dhanasekaran et al. 2010). Later, a new concept of drug-eluting embolic transarterial chemoembolization (DEB-TACE) using microspheres was introduced (Huppert 2011). This technique effectively combines enhanced local drug delivery and ischemic embolization effects (Huppert 2011). Therefore, the chemotherapeutic agent is tightly connected to the microspheres. The microspheres have been developed for transcatheter treatment of HCC to deliver higher doses of the chemotherapeutic agent and to prolong contact time with the tumor (Nam et al. 2016). First, they were available in sizes ranging between 500 and 300 µm to deliver a high amount of chemotherapeutic agent into the target tumor. In time, smaller sized microspheres were developed that can reach into smaller tumor feeding vessels with a potentially superior efficacy and increased necrosis of the target tissue (Sattler et al. 2018). Throughout the years, the development led to sizes ranging between 100 and 70 µm and finally the extremely small 40-µm microspheres. The size distribution allows for better steerable vessel occlusion that minimizes the undesirable side effects of a non-target embolization.

This retrospective study examined procedure-related complications in relation to the therapeutic response by evaluating pre-and post-treatment magnetic resonance imaging (MRI) and computed tomography (CT) scans after DEB-TACE with 40 µm-sized microspheres. In addition, radiological image histograms, classification, and further clinical parameters were evaluated.

## Materials and methods

### Study design

This retrospective study was conducted in a tertiary care university hospital and was approved by the local institutional review board. The requirement for informed consent was waived due to the retrospective nature of the study. The observation period was from May 2012 to May 2017.

### Patients

The study population consisted of 137 consecutive adult patients with HCC who received DEB-TACE with 40 µm-sized microspheres. The exclusion and inclusion criteria are shown in Table 1. Eighty-three patients were enrolled. The characteristics of the patients are summarized in Table 2 (women, 8; men, 75; mean age, 64.7 years; range 32–84 years). Forty-eight patients received an HCC diagnosis confirmed by histological examination. Thirty-five patients were diagnosed with HCC using at least 2 imaging modalities (CT, MRI, or ultrasonography) (*n* = 24) or by use of 1 liver imaging modality combined with an increased level of alpha-fetoprotein (AFP) (> 20 ng/ml) (*n* = 11) (Abbasi et al. 2012).

**Table 1:** Inclusion and exclusion criteria

Inclusion criteria	Exclusion criteria
Only the usage of 40 µm sized microspheres during DEB-TACE	Different particle size (100 or 75 µm)
Liver imaging before and after each treatment	Lipiodol application (conventional TACE)
No other simultaneous treatment	Missing follow-up investigation
– Therapy with sorafenib (Nexavar ™)	Different therapy directly after TACE
– Radioembolization	Liver transplantation shortly after TACE (median: 30.5 days)
– Surgical resection	Therapy with sorafenib (nexavar)
– Conventional TACE with lipiodol (c-TACE)	Missing arterial phase imaging in follow up investigation
– Other particle sizes	Extensive target lesion

*DEB-TACE* drug-eluting embolic transarterial chemoembolization, *c-TACE* conventional TACE

**Table 2:** Demographic characteristics and tumoral parameters of the study population (*n* = 83)

Age	64.7 years (32–84 years)	
Sex	75 male, 8 female	
Child–Pugh Score		
	*n*	%
A	45	54.2
B	33	39.8
C	5	6.0
ECOG Score		
	*n*	%
0	61	73.5
1	17	20.5
2	5	6
BCLC stages		
	*n*	%
0	4	4.82
A	42	50.60
B	30	36.15
C	1	1.20
D	6	7.23
Tumor extension		
Tumor size	36.78 ± 28.20 mm (minimum: 7.50 mm, maximum 172.10)	
Number of tumor lesions	138	
Number of lesions per patient	1.35 ± 0.688 (minimum: 1, maximum 4)	
	*n*	%
Unilobar	61	73.5
Bilobar	22	26.5
DEB-TACE re-treatment cycle		
	*n*	%
1	49	59.04
2	20	24.1
3	7	8.43
4	4	4.82
5	3	3.61
Cause	Amount of patients (*n* = 90)^1^	%
Alcohol intake	55	61
Hepatitis	8	9
-HBV B	2	
-HBV C	6	
Hemochromatosis	8	9
Autoimmune hepatitis	2	2
Nonalcoholic steatohepatitis	5	6
Unknown origin	8	9
Non cirrhotic liver	4	4

*ECOG* Eastern Co-operative Oncology Group, *BCLC* Barcelona Clinic Liver Cancer, *DEB-TACE* drug-eluting embolic transarterial chemoembolization, *HBV* hepatitis B virus, *HBC* hepatitis C virus

The indications for DEB-TACE treatment were bridging for transplantation in 49 (59%) cases, palliative treatment in 29 (35%) cases, and as a curative approach in 5 (6%) cases, as the size of the index tumor was not major at the time of diagnosis. Forty-two patients fulfilled the Milan criteria (a single tumor 5 cm or less in size or up to 3 tumors, each 3 cm or less in size, and no macroscopic vascular invasion (Mazzaferro et al. 2009)); 41 patients did not fulfill the criteria. The Barcelona Clinic Liver Cancer (BCLC) staging, which connects the disease stage, liver function, and health status to a certain treatment was also applied as shown in Table 2. Further information regarding the etiology of the underlining liver disease is also shown in Table 2. A total of 30 patients had received previous treatment before inclusion with a mean of 7.7 ± 8.0 months (range 1.1–37.9 months) having elapsed between the prior treatment and the DEB-TACE (Online Resource 1).

### DEB-TACE procedure

The DEB-TACE was performed by four interventional radiologists with more than 5 years of experience (mean, 16 years; range 11–25 years). The embolization was performed with a coaxial superselective, subsegmental technique using a 5F cobra or 5F sidewinder catheter, followed by the positioning of a microcatheter (Progreat 2.8, Tokyo, Japan), which was placed as close as possible to the arterial vessel supplying the tumor. One milliliter of Tandem Embozene™ (Boston Scientific, Marlborough, MA, USA) 40-µm microspheres was loaded with 50 mg doxorubicin and diluted in 4-ml ionic contrast medium (Solutrast™ 250 mg, Bracco, Milan, Italy). The radiologist used either 2-ml Tandem Embozene™ (with 100 mg doxorubicin) in a 10-ml syringe or 3-ml Tandem Embozene™ (with 150 mg doxorubicin) in a 15-ml syringe. For large tumors > 3 cm, or more than three nodules adding to a diameter > 3 cm, 3-ml vials of microspheres were used. The mixture was slowly injected using 1-ml syringes until blood flow stasis was achieved in the target vessel.

### Image evaluation

Image evaluation was performed using the software Mint Lesion™ for therapeutic response evaluation (Mint Medical, Dossenheim, Germany). The software allows for efficient workflow and supports radiologists in reporting the imaging studies. It applies the mRECIST criteria precisely by differentiating between complete response (CR), partial response (PR), stable disease (SD), and progressive disease (PD). CR is the disappearance of any intratumoral arterial enhancement in all target lesions.

PR is at least a 30% decrease in the sum of diameters of viable (contrast enhancement in the arterial phase) target lesions, taking reference the baseline sum of the diameters of target lesions.

PD is an increase of at least 20% in the sum of the diameters of viable (enhancing) target lesions, taking as reference the smallest sum of the diameters of viable (enhancing) target lesions recorded since the treatment started.

SD defines any cases that do not qualify for either PR or PD (Lencioni and Llovet 2010).

Therefore, it was essential to upload the pre-and post-interventional images and to detect the HCC lesions. In addition, the viable fraction of the HCCs with arterial enhancement was circumnavigated manually in each axial layer, and the size of the circumscribed area was calculated by adding the separate surfaces through Mint Lesion™ automatically. After this classification, Mint Lesion™ calculated the percentile alteration for all parameters in the histogram analysis (mean, entropy, kurtosis, mean value of positive pixels (MPP), skewness, standard deviation, uniformity of distribution of positive pixels (UPP), and uniformity), leading to the calculation of the tumor response (CR, PR, SD, PD) according to the mRECIST criteria (Online Resource 2).

The period between the CT or MRI scans before treatment and DEB-TACE was 23.0 ± 18.2 days. One hundred and forty-one TACE interventions were performed in 83 patients. This led to a total of 282 follow up examinations, which consisted of 255 CT scans and 27 MRI scans. The interval between re-treatments was in months: 4.4 ± 36.7. Criteria for re-treatment was the result of SD, PR or PD in the follow-up scans.

Despite applying the bolus tracking technique in CT imaging, the arterial phase did not always allow for precise detection of the hyperdense HCCs. The mRECIST criteria were obtained by measuring peak enhancement for the non-arterial cohort. It was necessary to use the portal venous phase in 13 CT (5.1%) scans (*n* = 13/255) and the venous phase in 6 CT (2.3%) scans (*n* = 6/255). In most cases, the HCCs were evaluated in the arterial phase (*n* = 236/255) (92.6%).

The analysis in Mint Lesion™ was based on two different mRECIST evaluations. First, as DEB-TACE is a locoregional procedure, a target-based evaluation of only treated lesions (classified as “target lesions”) was performed (target response, Table 3). Untreated lesions were declared to be “findings”. A second analysis was performed respecting all HCCs, regardless of the status of treatment (overall response, Table 3).

**Table 3:** Results for both analysis

	Target response		Overall response		Comparison of target response and overall response
	*n*	%	*n*	%	
CR	56	39.72	47	33.33	–
PR	33	23.40	37	26.24	+
SD	46	32.62	50	35.46	+
PD	6	4.26	7	4.97	+
RR (CR + PR)	89	63.12	84	59.57	
DCR (CR + PR + SD)	135	95.74	134	95.04	

*SD* stable disease, *PD* progressive disease, *CR* complete response, *PR* partial response, *RR* response rate, *DCR* disease control rate

### Safety profile

All adverse events (AE) were graded for all 141 DEB-TACE interventions and the use of the investigational product according to the Common Terminology Criteria for Adverse Events v 5.0. The safety evaluation was performed by examining if an AE was procedure-related or product related. A serious AE (SAE) based on the FDA’s regulatory definition denotes an AE that results in a health outcome of death, disability, or hospitalization is life-threatening, or requires intervention to prevent harm (Moore et al. 2007). Death within 30 days after the DEB-TACE intervention was regarded as an SAE. Hospitalization for longer than 3 days was considered as prolongation.

Post-embolization syndrome (PES) was defined as a DEB-TACE-related side effect. PES includes liver enzyme abnormalities, fever, abdominal pain, vomiting, and nausea (Lencioni et al. 2016).

### Statistical analysis

All calculations were performed with the statistical software SPSS™ (Version 24, IBM, Armonk, NY, USA). The data are presented as median and standard deviation. The compiled parameters from the histogram analysis and laboratory data, which were collected before and after each treatment cycle, were analyzed using the Wilcoxon test. The correlation between the longitudinal axis and volume was evaluated using Spearman’s test. The outcome parameters were overall survival (OS), progression-free survival (PFS), and time to progression (TTP). All parameters were calculated with the Kaplan–Meier method. The overall survival (OS) was calculated from the date of the first DEB-TACE procedure to death/latest follow-up. PFS was defined as the period from the first treatment until objective tumor progression (an increase of at least ≥ 20% in the viable target lesion) or death (Nakamura et al. 2012). TTP was defined as the period from the first treatment until objective tumor progression (an increase of at least ≥ 20% in the viable target lesion) not considering the event “death” (Saad 2008). Both PFS and TTP were determined after each treatment cycle.

## Results

### DEB-TACE

The mean number of treatments per patient was 1.7. The average amount of doxorubicin in each intervention was 75.00 ± 53.25 mg (minimum, 2.2 mg; maximum, 150.00 mg).

### Survival

**Fig. 1:**
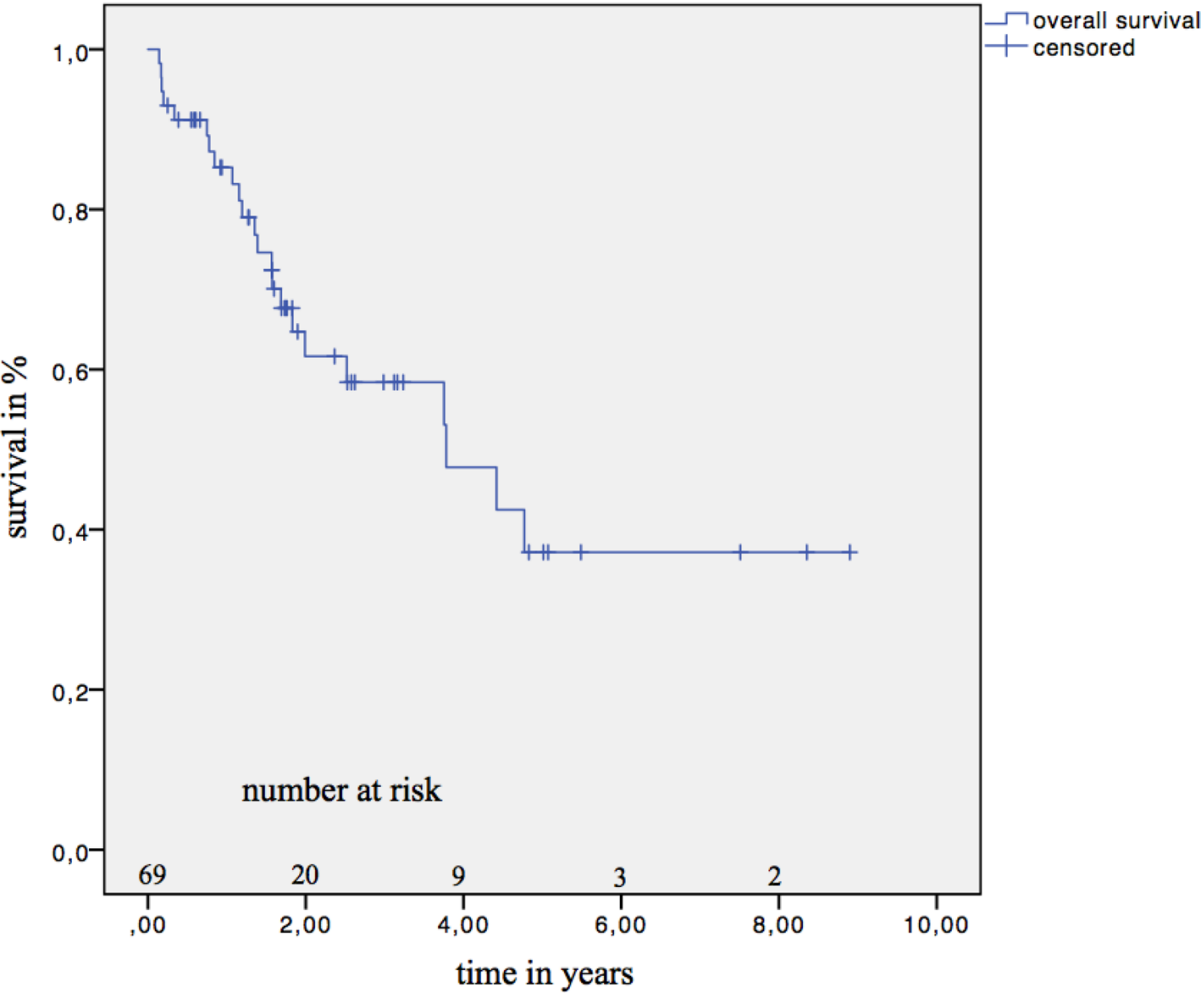
Overall survival

A total of 28 patients (33.7%) died after HCC was diagnosed (range, 1.7–57.3 months). In this group, 13 patients with CR (46.43%), 7 with SD (25.00%), 6 with PR (21.43%), and 2 with PD (7.14%) died. Overall six patients were diagnosed with PD. Despite the fact, that 49 patients were submitted to TACE for bridging, only 14 patients received a liver transplant (reasons for drop-out are listed in Online Resource 3). The period from the listing to receiving the organ was 9.2 ± 3.9 months. Despite receiving a liver transplant, four patients died. One patient that received a liver transplant had an extra hepatic relapse (pulmonary and lymphogenous metastasis) 2 years later. Therapy with sorafenib was started but was not successful. Among the 69 patients who did not receive a liver transplant, the OS (Fig. 1) was 83.2% (standard error [SE] 5.1%) after 1 year, 61.6% (SE 7.4%) after 2 years, 58.4% (SE7.7%) after 3 years, and 47.8% (SE 9.2%) after 4 years. The median OS could not be calculated, as 30.7% of the patients died and the median OS can only be calculated when half the patients die.

**Fig. 2:**
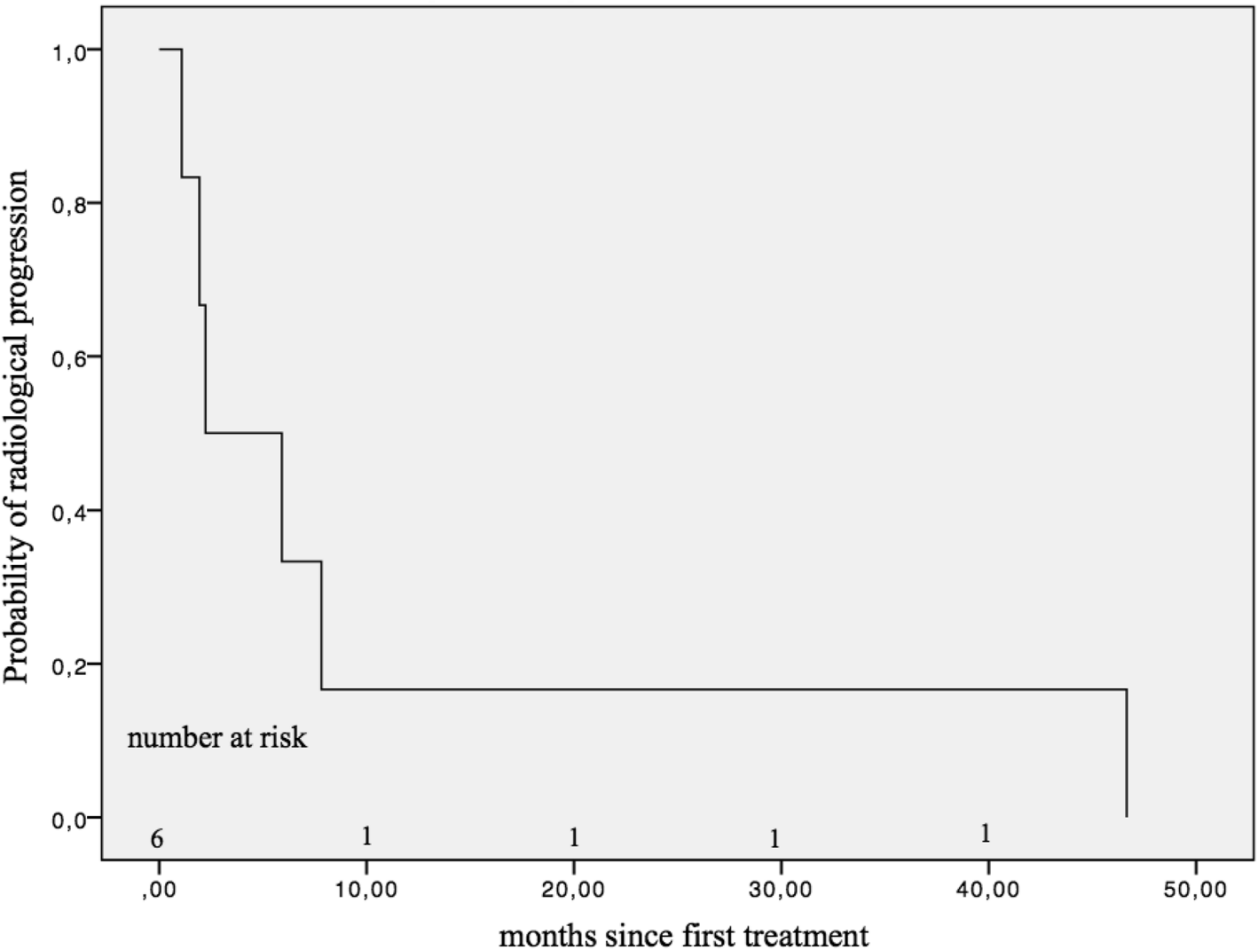
Progression-free survival

**Fig. 3:**
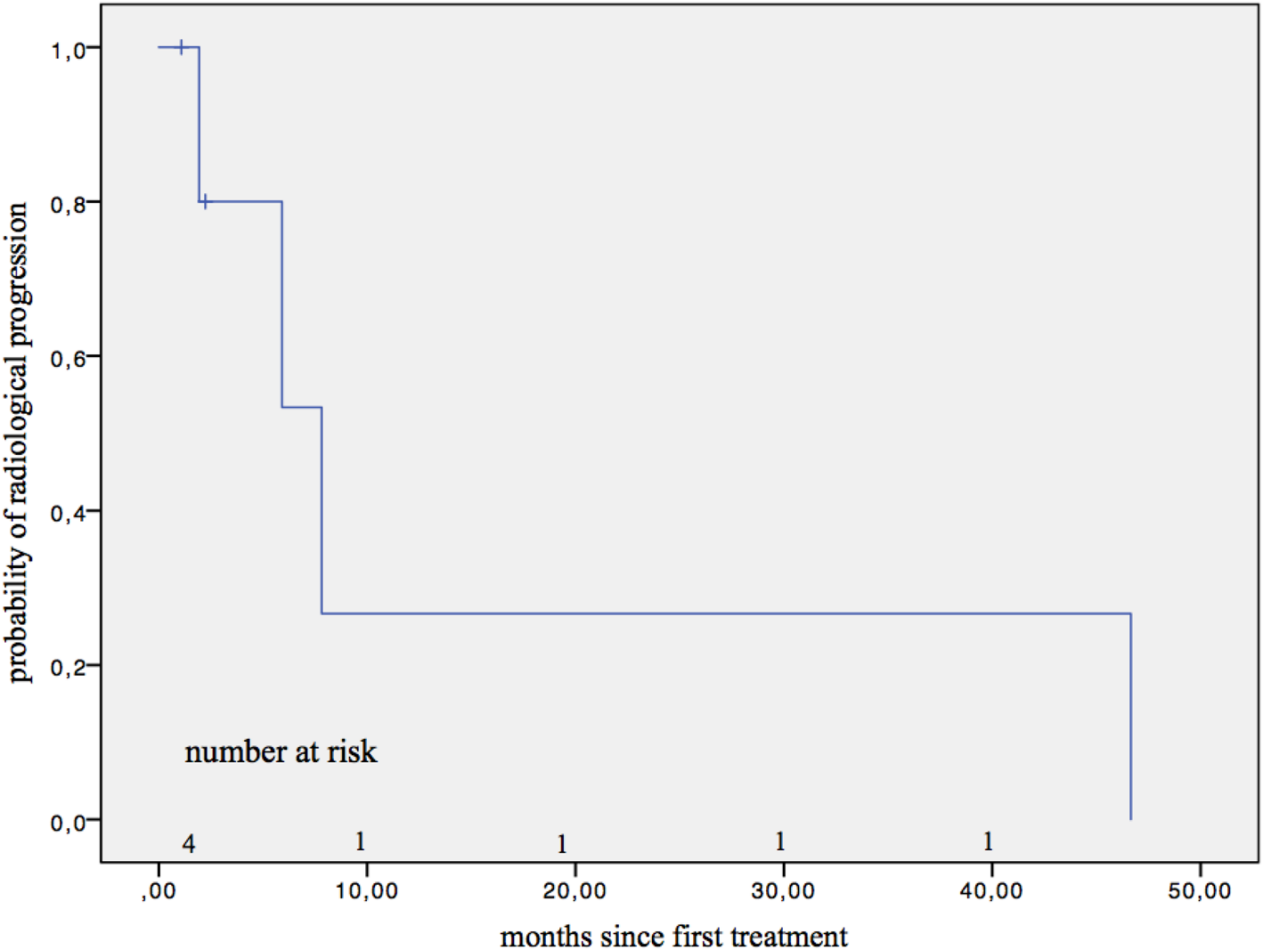
Time to progression

Throughout all five re-treatment cycles in 83 patients (Online Resource 4) with totally 141 re-treatment cycles (Online Resource 5), 6 patients had tumor progression according to the mRECIST criteria (after first cycle: *n* = 2; after second cycle: *n* = 1; third cycle: *n* = 2; fourth cycle: *n* = 1; fifth cycle: *n* = 0). Radiological progression was detected in six patients (4.26%), with a median PFS of 2.23 months (SE 2.43 months) (Fig. 2). Two of these patients died at the end of the follow-up period, which resulted in a median TTP of 7.81 months (SE 2.49 months) (Fig. 3).

Although the exclusion and inclusion criteria (Tab.1) were strictly endorsed, five patients in our cohort had advanced stages of HCC. They were relatively young at the time of diagnosis (58–64 years), and none of these patients developed PD during the DEB-TACE therapy. All tumors responded to DEB-TACE, and three patients even reached CR status. Four of the five patients with advanced liver disease died. The time range between the last TACE treatment and the date of death was 113.0 ± 93.1 days. The death was caused by decompensated cirrhotic liver disease and its consequences.

### Safety

AEs were reported in 25 cases, documented in Online Resource 6. A groin hematoma (0.7%) and a hypertensive crisis (0.7%), which occurred during the procedure, were found to be unrelated to the use of 40-µm microspheres. Two female patients developed acute pancreatitis after DEB-TACE treatment. In both cases, prolongation of the hospital stay was necessary. It remains unclear whether the pancreatitis was caused by the use of the 40 µm-sized microspheres. PES with prolongation of the hospital stay was observed in 12 cases (Online Resource 7). These patients had a large tumor burden and received, therefore, a high amount of embolic agent thus resolving in a high doxorubicin amount.

One SAE was recorded due to death from acute liver failure 16 days after the DEB-TACE intervention. The longitudinal axis of the tumor decreased from the baseline to the follow-up CT examination by > 56% (from 138.60 to 61.50 mm). The high tumor reduction led to tumor lysis syndrome with respiratory, kidney, and, finally, liver failure. The SAE rate was 10.64%, considering the one case of death, the 12 cases of PES, and the two cases of acute pancreatitis.

### Radiological tumor response

The therapy response rate was determined with two different methods. First, a “target response analysis” was performed considering only the treated lesions, and second, an analysis that included all HCC lesions. The results differed in 18 cases as shown in Table 3.

The response rate according to the mRECIST criteria for the target response analysis (Table 3) was in most cases considered as either CR (39.72%, *n* = 56/141) or SD (32.62%, *n* = 46/141). The response rate (CR + PR) was 63.12%. The disease control rate (CR + PR + SD) was 95.74%. The analysis also included an evaluation of whether ascites and/or portal venous thrombosis was present at each investigational time point as shown in Online Resource 8. In this study, the incidence of ascites increased between the pretreatment and post-treatment imaging for the first and second DEB-TACE cycles. The incidence of portal venous thrombosis remained the same in the pre- and post-treatment scans. In three cases, portal vein thrombosis was due to tumoral infiltration.

**Fig. 4:**
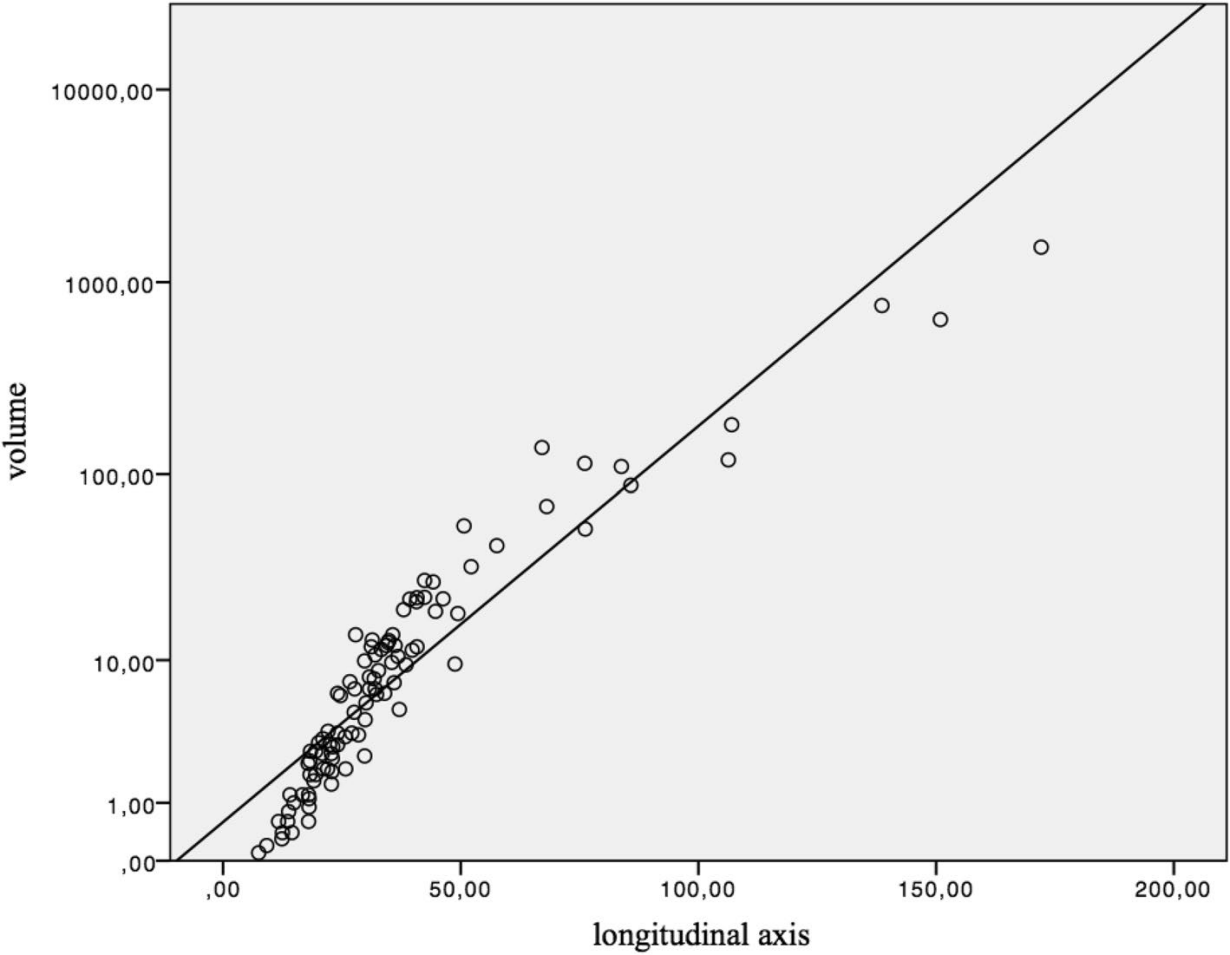
Correlation between volume and the longitudinal axis

The correlation between the longitudinal axis and volume was examined. The r value was 0.954, which indicated a significant correlation between the longitudinal axis and volume (p < 0.001) (Fig. 4).

The reduction of volume (46.1 ± 181.8 to 28.0 ± 146.3 ml), longitudinal axis (36.8 ± 28.21 to 19.6 ± 30.9 mm), and short axis (23.2 ± 17.1 to 11.0 ± 17.2 mm) in the treated lesions before and after each treatment was statistically significant, at *p* = 0.0001.

The histogram analysis showed alterations in entropy, uniformity, MPP, UPP, and minimal and maximal density (all, *p* < 0.03). For kurtosis, middle density, standard deviation, and skewness, the data before and after each treatment cycle did not change significantly.

## Discussion

In this retrospective analysis, the main focus was to examine the safety and therapy response rate of 40-µm microspheres for the treatment of HCC by investigating the tumor characteristics, such as the longitudinal axis, volume, and the parameters included in the histogram analysis after the treatment.

The therapy response rate was estimated by the use of the medical software mint lesion™, which also included a histogram analysis. To our knowledge, this is the first investigation in patients with HCC, that contained this kind of analysis. We determined that a correlation between longitudinal axis and volume existed and that a decrease in the longitudinal diameter also resulted in a decline of volume.

This analysis did not include a comparison group (e.g., different particle sizes or conventional TACE group); therefore, it cannot be claimed with certainty that the study outcomes are specific to the use of 40 µm-sized microspheres. Recently, Greco et al. demonstrated in a retrospective study the safety profile and response rate of 40-µm doxorubicin-loaded microspheres. Their study population consisted of 48 patients with early intermediate HCC, who showed an objective response rate of 72.6% according to mRECIST (Greco et al. 2017). In comparison, this study population was larger, with 83 patients, but achieved comparable therapeutic success, with an objective therapy response rate of 63.12%.

In 2011, Huppert et al. stated that the grade and duration of PES symptoms depend on the size of the tumor and surrounding liver tissue affected by TACE and on the amount of embolic material applied (Huppert 2011). In the animal study of Dinca et al. in which small microspheres of 30–60 µm provided a very effective distal occlusion and distribution of microspheres in the embolized territory than microsphere of 50–100 µm. (Dinca et al. 2012) The usage of tightly calibrated microspheres in the size of 40 µm also allows a deeper penetration of the nourishing tumor vessel and substantially accumulates the chemotherapeutic agent doxorubicin only in the tumor itself. Malagari et al. declared that distal embolization is desirable to avoid hypoxia-induced neoangiogenesis. (Malagari et al. 2014)

Our study findings support this claim, as the 12 patients who developed PES were the ones who received a high dose of doxorubicin (95.5 ± 50.8 mg) and consequently also a high amount of embolic agent. This group of patients had large HCC nodules, but they were also the ones with a good therapy response rate (CR = 5/12, PR = 3/12, SD = 4/12). Prajapati et al. demonstrate that with different sizes of microspheres larger areas of necrosis may be due to the increased surface area of smaller beads inducing a greater burst release of doxorubicin within the tumor (Prajapati et al. 2014).

The SAE rate in this analysis was 10.64%, including the 12 patients who developed PES, 1 case of death 16 days after the DEB-TACE cycle, and 2 cases of acute pancreatitis. Table E6 lists the symptoms that were monitored after 141 DEB-TACE interventions. In our patient cohort, only two patients developed a liver abscess with increased liver and infection parameters.

Dhamija et al. stated that biliary complications are uncommon (0.87%) and that the biliary ducts have an exclusive blood supply from the hepatic artery branches; as a consequence of hepatic artery embolization, it is possible that an inadvertent occlusion may occur during TACE (Dhamija et al. 2015). None of these biliary complications were monitored in this study. However, two cases of acute pancreatitis after DEB-TACE were observed. The pancreatitis could have been caused by reflux of loaded microspheres due to potential communication channels around the common bill duct and liver hilum between the hepatic and pancreaticoduodenal arteries.

Interestingly, a prospective trial by Lee et al. found that 100–300-µm microspheres (DC Bead; Biocompatible, Farnham, UK) had excellent efficacy, but a relatively high overall incidence of PES at 67.6% in that cohort. Furthermore, in 19.7% of the patients, imaging showed prominent biliary injury (Lee et al. 2015). In comparison, in this study, the PES rate was fairly low, which could not only be related to the study’s retrospective nature, but could also indicate a tendency of lower complications due to the application of smaller sized microspheres.

The limitations of this study included its retrospective character and the monocentric implementation that led to an unavoidable selection bias of patients. Moreover, no control group was enrolled. Conventional TACE (c-TACE) with lipiodol emulsion is still considered the gold standard; therefore, a comparison between 40-µm DEB-TACE and c-TACE is recommended to accumulate additional clinical evidence.

## Conclusion

In conclusion, this retrospective analysis supports the hypothesis that a high response rate in the treatment of HCC can be achieved using small-sized microspheres, which allow a deeper penetration of the nourishing tumor vessel. This technique also minimizes the undesirable side effects of a false embolization.

## Electronic supplementary material

Below is the link to the electronic supplementary material.

Supplementary material 1 (DOCX 109 kb)

## Data Availability

The data that support the findings of this study will be made available after sponsor approval (Friedrich-Schiller-University Jena, Germany). In particular, individual participant data that underlie the results reported in this article (text, tables, figures, appendices), and other patient data will be available strictly for scientific reasons and only on demand. Source data will be made available especially for systematic reviews and meta-analysis.

## Abbreviations

DEB: Drug-eluting beats
TACE: Transarterial chemoembolization
HCC: Hepatocellular carcinoma
CR: Complete response
PR: Partial response
SD: Stable disease
PFS: Progression-free survival
TTP: Time to progression
SAE: Serious adverse event
MRI: Magnetic resonance imaging
CT: Computed tomography
AFP: Alpha-fetoprotein
PD: Progressive disease
MPP: Mean value of positive pixels
UPP: Uniformity of distribution of positive pixels
mRECIST: Modified response evaluation criteria in solid tumors
AE: Adverse events
PES: Post-embolization syndrome
c-TACE: Conventional TACE

## Code availability

The software application used for the image evaluation of therapeutic response was Mint Lesion™ (Mint Medical, Dossenheim, Germany).
