# Supplementary material for: Response rate and safety in patients with hepatocellular carcinoma treated with transarterial chemoembolization using 40-µm doxorubicin-eluting microspheres
Source: J Cancer Res Clin Oncol. 2020 Sep 2;147(1):23–32. doi: 10.1007/s00432-020-03370-z (PMC7810642; doi:10.1007/s00432-020-03370-z)
Supplement: Supplementary file 1 — Supplementary material 1 (DOCX 109 kb) [file 432_2020_3370_MOESM1_ESM.docx]

**Electronic Supplementary Material (ESM):**

This appendix has been provided by the authors to give readers additional information about their work.

Supplement to manuscript:

**Response Rate and Safety in Patients with Hepatocellular Carcinoma Treated with Transarterial Chemoembolization Using 40-µm Doxorubicin Eluting Microspheres**

| Online Resource 1: Previous Treatment (n=30) | | |
| --- | --- | --- |
|  | n | % |
| DEB-TACE | 18 | 60.0 |
| SIRT | 3 | 10.0 |
| RFA | 6 | 20.0 |
| surgical treatment | 3 | 10.0 |
| -atypical liver resection | 2 | 6.7 |
| -hemihepatectomy | 1 | 0.3 |

Online Resource 2: Explanation for mean, skewness and kurtosis in the histogram analysis
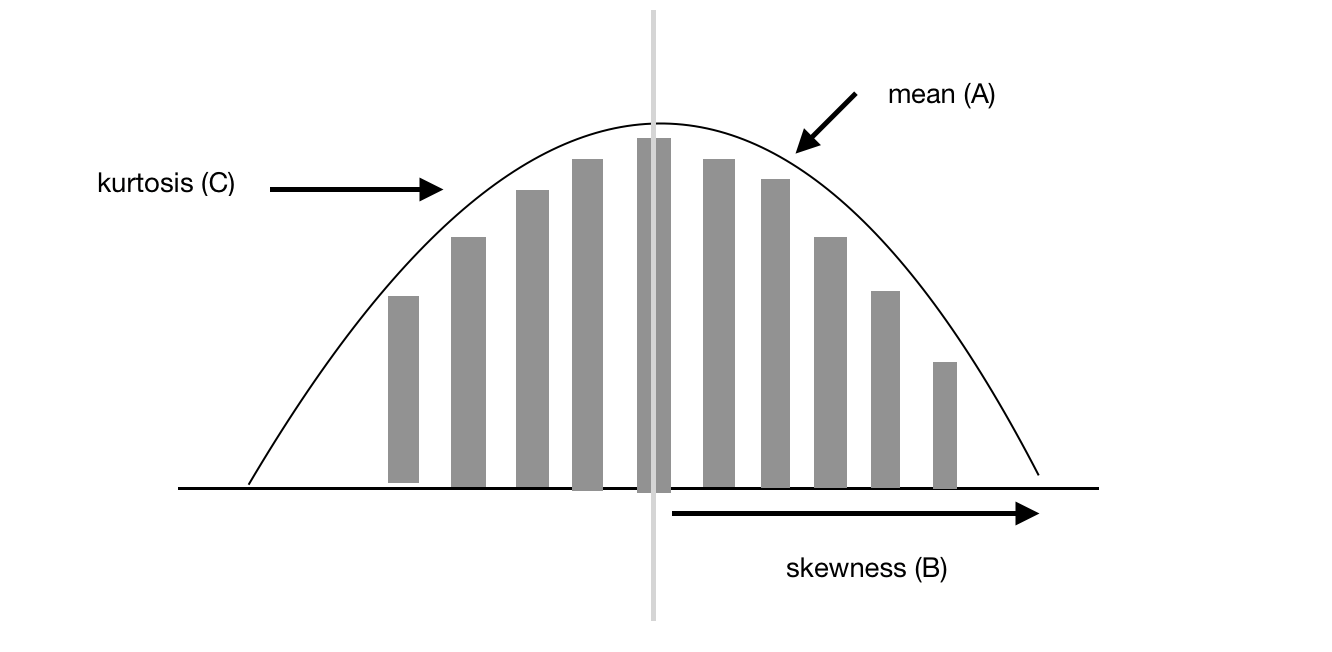


Explanation:

Mean (A) is the average value of pixels within the circumnavigated lesion. The standard deviation (SD) is a measurement of how much variation or dispertion exists from the average (mean value). A low SD indicated that the data points tend to be very close to the mean in conclusion a high SD indicated that the data points are spread out over a large range. Scewness (B) is a measurement of the asymmetry of the histogram. It can be positive or negative. A negative scew indicated that the tail on the left side of the histogram is longer that the right side. A positive scew indicates that the tail on the right side is longer than the left side. A zero value indicated that the values are evenly distributed on both sides of the mean. Kurtosis is a measure of the peakedness of the histogram. The kurtosis (C) value can be positive or negative. A positive kurtosis indicated a histogram that is more peaked than a Gaussian (normal) distribution. A negative kurtosis indicated that histogram is flatter than a Gaussian distribution. The mean of positive pixels (MPP) is a measurement that only considers pixels greater than zero and therefore reduces the impact of dark objects.(1) Entropy represents the irregularity or complexity of pixel intensity in space. (2). Uniformity describes the steadiness of grey level distribution (1). UPP is uniformity of distribution of positive pixels.

| Online Resource 3: Reasons for not receiving a liver transplant | |
| --- | --- |
| n | reason for drop-out |
| 14 | received LTx |
| 3 | loss of follow up information |
| 11 | lack of vital tumor lesion in the follow up CT/MRI scan |
| 1 | developed another tumor disease |
| 5 | stable disease in follow up scan |
| 2 | deterioration of the general condition |
| 6 | tumor progression in the follow up scan |
| 7 | death |

LTx= liver transplantation, CT= Computed tomography, MRI= Magnetic Resonance Imaging

| Online Resource 4: Therapy Response according to mRECIST in patients (n=83) | | | | | |
| --- | --- | --- | --- | --- | --- |
|  | **1** | **2** | **3** | **4** | **5** |
| **CR** | 23 | 12 | 1 | 3 |  |
| **PR** | 11 | 4 |  |  | 1 |
| **PD** | 2 | 1 | 1 |  |  |
| **SD** | 13 | 3 | 5 | 1 | 2 |

CR= Complete response, PR= partial response, PD= progressive disease, SD= stable disease, mRECIST= modified Response Evaluation Criteria in Solid Tumors

| Online Resource 5: Therapy Response according to mRECIST for 141 treatment cycles | | | | | |
| --- | --- | --- | --- | --- | --- |
|  | **1** | **2** | **3** | **4** | **5** |
| **CR** | 33 | 18 | 2 | 3 | 0 |
| **PR** | 23 | 7 | 2 | 0 | 1 |
| **PD** | 2 | 1 | 2 | 1 | 0 |
| **SD** | 25 | 8 | 8 | 3 | 2 |

CR= Complete response, PR= partial response, PD= progressive disease, SD= stable disease, mRECIST= modified Response Evaluation Criteria in Solid Tumors

| Online Resource 6: Adverse Events (AE) (n=25/141) | | |
| --- | --- | --- |
|  | n | % |
| pain | 5 | 3.6 |
| increased liver parameters | 18 | 12.7 |
| increased infect parameters | 1 | 0.7 |
| nausea | 1 | 0.7 |

| Online Resource 7: Post-embolization syndrome (n=12/141) | | | | | | | |
| --- | --- | --- | --- | --- | --- | --- | --- |
| intervention (n) | abdominal pain | nausea/vomiting | fever | abscess | increased infect parameter | increased liver parameters | prolongation of the hospital stay ^1^ |
| 1 | x | x |  |  |  |  |  |
| 2 |  |  | x |  | x |  | x |
| 3 |  |  |  |  | x | x | x |
| 4 | x |  | x |  | x | x | x |
| 5 |  |  |  | x ^2^ | x | x | x |
| 6 | x | x |  |  |  | x | x |
| 7 |  |  | x |  |  |  | x |
| 8 |  |  | x |  | x |  | x |
| 9 |  |  | x |  |  | x | x |
| 10 |  |  |  |  | x | x | x |
| 11 | x |  |  | x ^2^ |  |  | x |
| 12 |  |  |  |  |  | x | x |

^1^ duration of the hospital stay was > 3 days

^2^ a drainage was not necessary

| Online Resource 8: Appearance of ascites and portal venous thrombosis | | | | | | | | |
| --- | --- | --- | --- | --- | --- | --- | --- | --- |
|  | ascites, pretreatment imaging |  | ascites, post treatment imaging |  | portal venous thrombosis, pretreatment imaging |  | portal venous thrombosis, post treatment imaging |  |
| DEB-TACE cycle | n | % | n | % | n | % | n | % |
| 1 (n=49) | 26 | 53.1 | 33 | 67.3 | 7 | 14.3 | 7 | 14.3 |
| 2 (n=20) | 14 | 70.0 | 16 | 80.0 | 2 | 0.1 | 2 | 0.1 |
| 3 (n=7) | 7 | 100.0 | 6 | 85.7 | 0 | 0 | 0 | 0 |
| 4 (n=4) | 3 | 75.0 | 3 | 75.0 | 0 | 0 | 0 | 0 |
| 5 (n=3) | 1 | 33.3 | 1 | 33.3 | 0 | 0 | 0 | 0 |

DEB-TACE= drug eluting beads transarterial chemoembolization

References:

(1)Davnall, F., C. S. Yip, G. Ljungqvist, M. Selmi, F. Ng, B. Sanghera, B. Ganeshan, K. A. Miles, G. J. Cook and V. Goh (2012). "Assessment of tumor heterogeneity: an emerging imaging tool for clinical practice?" Insights Imaging 3(6): 573-589.

(2)Haider, M. A., A. Vosough, F. Khalvati, A. Kiss, B. Ganeshan and G. A. Bjarnason (2017). "CT texture analysis: a potential tool for prediction of survival in patients with metastatic clear cell carcinoma treated with sunitinib." Cancer Imaging 17(1): 4.
